# A large nabothian cyst causing chronic urinary retention: A case report

**DOI:** 10.1097/MD.0000000000019035

**Published:** 2020-02-07

**Authors:** Zhao Wu, Bingyu Zou, Xun Zhang, Xue Peng

**Affiliations:** a Department of Obstetrics and Gynecology, Sichuan Academy of Medical Sciences & Sichuan Provincial People's Hospital, Chengdu 610072; b Department of Obstetrics and Gynecology, West China Second University Hospital, Sichuan University, Chengdu 610041; c Key Laboratory of Birth Defects and Related Diseases of Women and Children, Sichuan University, Ministry of Education, Chengdu 610041, China.

**Keywords:** cervix, chronic urinary retention, nabothian cyst

## Abstract

**Rationale:**

Nabothian cysts are mucus-filled cervical cysts that are usually asymptomatic unless they become very large. Chronic urinary retention is the persistent inability to empty the bladder despite maintaining an ability to urinate. Chronic urinary retention caused by a large, deep nabothian cyst has not been reported previously.

**Patient concerns:**

A 46-year-old woman presented with chronic urinary retention and a cervical cyst that gradually increased in size.

**Diagnosis:**

Based on histopathological evidence, our patient was diagnosed with a nabothian cyst.

**Interventions:**

A hysterectomy was performed.

**Outcomes:**

The urinary symptoms of the patient resolved after she performed clean, intermittent self-catheterizations for 5 days after the operation. She was discharged on postoperative day 6.

**Lessons:**

Large nabothian cysts are rare but may account for some unusual symptoms including unexplained urinary difficulties in women. We recommend treating symptomatic nabothian cysts with local cystectomies or hysterectomies.

## 1 Introduction

Nabothian cysts are mucus-filled cysts that occur on the surface of the cervix. They are usually 0.2 to 0.3 cm in diameter, but they can exceed 1 cm in diameter. In most cases, nabothian cysts reflect the physiological changes in the cervix, but they are sometimes related to chronic cervicitis. Treatment is usually unnecessary, and nabothian are asymptomatic unless they become very large.^[1,2]^

Urinary retention is the inability to completely empty the bladder of urine, and chronic urinary retention (CUR) is the persistent inability to completely empty the bladder despite maintaining an ability to urinate. CUR results in elevated postvoid residual urine volumes (PVRs). Two common causes of CUR are bladder muscle dysfunction and urinary tract obstructions.^[3]^

Herein, we report the case of a 46-year-old woman who presented with a large, deep nabothian cyst that contributed to CUR.

## 2 Case report

A 46-year-old woman (gravida 3, para 2) was admitted to our gynecology clinic. She reported that an ultrasonography assessment performed 5 years earlier revealed a cervical cyst with a 3-cm diameter. However, she did not report experiencing any specific symptoms at the time. Follow-up revealed that the cyst had gradually increased in size. During the previous 2 years, she experienced gradually worsening urinary difficulties in addition to dysmenorrhea and menorrhagia but did not seek any further medical attention.

**Figure 1:**
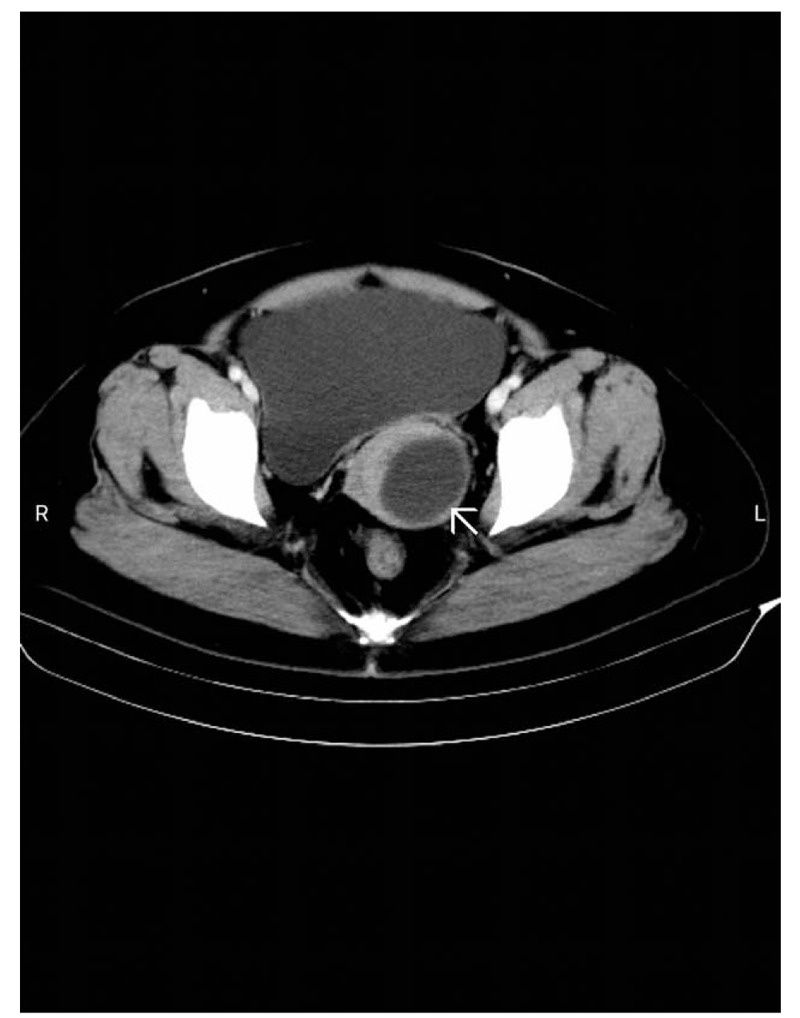
Pelvic computed tomography scan showing an enlarged and distended bladder and a cervical cyst (arrow).

Pelvic examinations revealed a uterus with normal bilateral adnexa, but the size was equivalent to that of a uterus during the 12th week of pregnancy. The enlarged cervix was palpable during a rectovaginal examination. Transvaginal ultrasonography confirmed that the uterus was abnormally large and showed that it had a heterogeneous myometrial echotexture and an unremarkable endometrium with a 5 cm × 4.5 cm anechoic cyst in the posterior cervical wall. Pelvic computed tomography revealed an enlarged and distended bladder and bilaterally dilated ureters and confirmed the presence of a cervical cyst with a 5-cm diameter (Fig. 1).

An indwelling Foley catheter was inserted, and 900 ml of urine was drained. Subsequent laboratory tests showed that our patient's hemoglobin level was 52 g/l (normal range, 110–160 g/l) and that her tumor biomarker carcinoembryonic antigen 125 level was 74.5 U/ml (normal range, <35 U/ml). She underwent a blood transfusion to treat her anemia. Cervical and endometrial biopsy samples tested negative for atypia.

**Figure 2:**
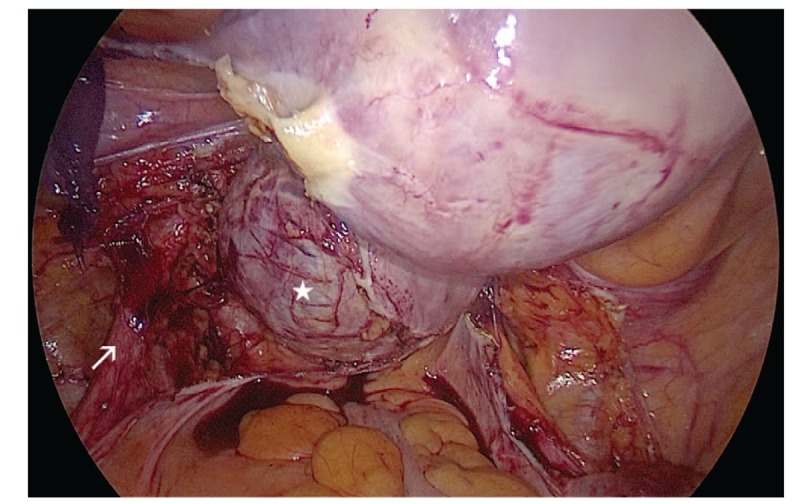
Intraoperative image showing the cyst (star) and the left ureter (arrow).

**Figure 3:**
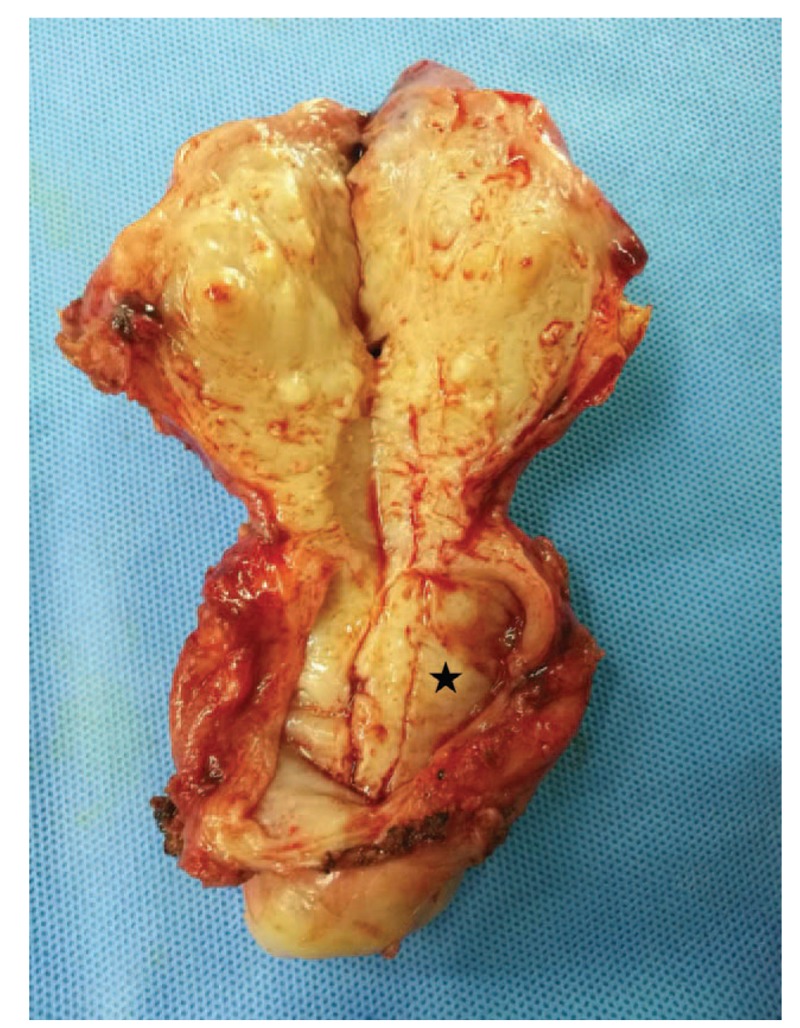
Image of the uterus showing the dissected cervical nabothian cyst (star).

Because she had adenomyosis and severe anemia, a laparoscopic hysterectomy was selected as the best treatment option. As shown in an intraoperative image (Fig. 2), after dissection of the posterior leaf of the broad ligaments and uterosacral ligaments, we identified the ureters; the cyst was visible, and the cervix was pushed to the right lateral anterior side by the cyst. The cyst was filled with mucous fluid and was located in the posterior left lateral wall of the cervix (Fig. 3). Additionally, we noted diffuse uterine enlargement and thickened myometrium with scattered nodules and small hemorrhagic foci.

**Figure 4:**
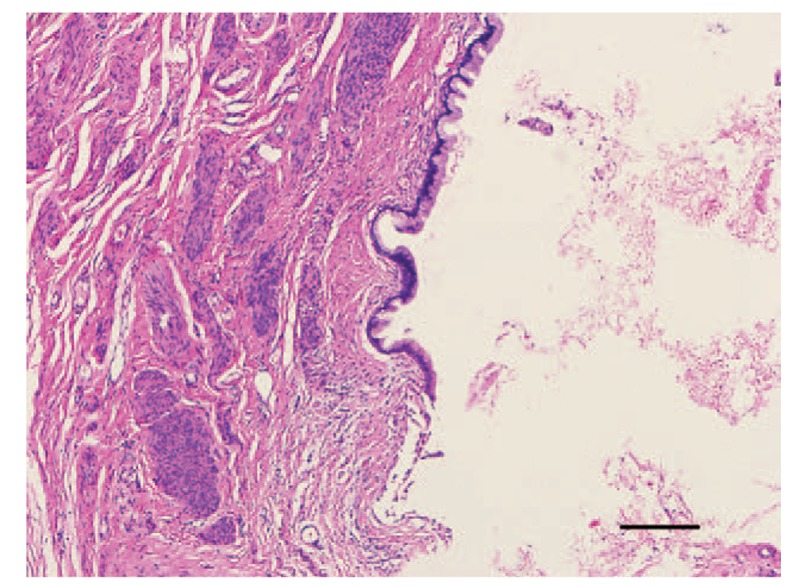
Histopathological images of the nabothian cyst showing the columnar epithelium without atypia. Scale bar 100 μm (hematoxylin and eosin staining; magnification, 200×).

Histopathological examinations of recovered cyst tissue showed that the cyst wall was lined with columnar epithelium, and endometrial glands and stroma were present deep within the myometrium (Fig. 4). We, therefore, diagnosed our patient with nabothian cyst and adenomyosis. She performed clean intermittent self-catheterizations for 5 days after the operation. By postoperative day 6, her urinary symptoms were completely resolved, and she was discharged from the hospital. No evidence of recurrence was observed during the 12 months of follow-up.

Our patient gave written informed consent for the publication of her medical history and images.

## 3 Discussion

To the best of our knowledge, this is the first case report to describe a large, deep nabothian cyst contributing to CUR. Few existing case reports describe the treatment of large, symptomatic nabothian cysts, although transvaginal or laparoscopic cystectomies are generally performed to avoid unnecessary hysterectomies.^[4,5]^ In our patient's case, due to the concurrent presence of adenomyosis and severe anemia, hysterectomy was considered as the best treatment option after discussion with the patient. This treatment successfully resolved her CUR.

CUR results in urinary accumulation that leads to adverse clinical outcomes if left untreated. The PVR is the metric used to quantify urinary retention levels. Although no single PVR value can serve as a universal definition of CUR, the American Urological Association defines CUR as a PVR of >300 ml.^[6]^ There are many potential causes of urinary retention in women, and they can be subdivided into anatomic and functional subgroups. Anatomic causes can include obstructions resulting from pelvic organ prolapse, gynecological tumors, and intrinsic urethral lesions. Moreover, anatomic obstructions may arise from iatrogenic procedures such as anti-incontinence or gynecological surgeries. Functional causes include abnormal contraction of the periurethral muscle and failure to relax the muscles surrounding the urethra or bladder neck prior to urination.^[7]^

Nabothian cysts are caused by squamous epithelium obstructing the cervical crypt orifices.^[8]^ Nabothian cysts can occur in any part of the cervix, but it is generally large, deep nabothian cysts that produce cervical swelling, as observed in our patient. Nabothian cysts are usually asymptomatic and mostly benign. Treatment is usually unnecessary. However, as nabothian cysts increase in size, symptoms may gradually emerge due to compression of the surrounding organs. For example, compression of the rectum can result in abnormal defecation and tenesmus.^[9]^ Nabothian cysts that originate from the anterior lip of the cervix can cause hematometra and recurrent lower abdominal pain.^[10]^

Large nabothian cysts and nabothian cysts occurring in clusters, especially those located deep in the cervix, and they should be distinguished from minimal-deviation adenocarcinoma (MDA), which is a rare mucin-producing cervical adenocarcinoma. Patients with MDA usually present with abnormal watery discharges from the vagina. Magnetic resonance imaging is used to distinguish MDA from typical nabothian cysts.^[11]^

In adult women, uterine fibroids are the most common benign tumors that compress the urethra, impair urine flow, and cause CUR. Several case reports claimed that the surgical removal of uterine fibroids can alleviate urinary retention problems.^[12,13]^ The mechanism by which a nabothian cyst caused CUR in our patient may be similar to the mechanism by which uterine fibroids cause CUR. A nabothian cyst may obstruct the bladder's outlet by displacing the cervix and thereby compressing the urethra or the bladder neck.^[13]^ Another potential pathophysiological mechanism involves the mass causing radiculopathy by compressing the pudendal and sacral nerves and thus, interrupting the innervation of the detrusor muscle.^[14]^

In conclusion, this case report shows that large, deep nabothian cysts can cause CUR in women. Therefore, although large nabothian cysts are rare, they should be considered as potential causes of pelvic masses and urinary symptoms in women. Nabothian cysts can be distinguished from other mucus-producing cervical malignancies on the basis of symptomatology, preoperative examination results, and imaging techniques such as magnetic resonance imaging.

## Author contributions

**Conceptualization:** Zhao Wu, Xue Peng.

**Data curation:** Bingyu Zou, Xue Peng.

**Formal analysis:** Zhao Wu, Bingyu Zou, Xue Peng.

**Funding acquisition:** Zhao Wu, Xue Peng.

**Investigation:** Zhao Wu, Xun Zhang.

**Methodology:** Bingyu Zou, Xue Peng.

**Project administration:** Zhao Wu.

**Resources:** Zhao Wu. Bingyu Zou, Xun Zhang.

**Supervision:** Bingyu Zou, Xue Peng.

**Writing – original draft:** Zhao Wu.

**Writing – review & editing:** Zhao Wu, Xue Peng.
